# Supplementary material for: A multi-platform reference for somatic structural variation detection
Source: Cell Genom. 2022 Jun 8;2(6):100139. doi: 10.1016/j.xgen.2022.100139 (PMC9903816; doi:10.1016/j.xgen.2022.100139)
Supplement: Document S1. Figures S1–S4 [file mmc1.pdf]

Cell Genomics, Volume *2*

Supplemental information

A multi-platform reference for somatic structural variation detection

Jose Espejo Valle-Inclan, Nicolle J.M. Besselink, Ewart de Bruijn, Daniel L. Cameron, Jana Ebler, Joachim Kutzera, Stef van Lieshout, Tobias Marschall, Marcel Nelen, Peter Priestley, Ivo Renkens, Margaretha G.M. Roemer, Markus J. van Roosmalen, Aaron M. Wenger, Bauke Ylstra, Remond J.A. Fijneman, Wigard P. Kloosterman, and Edwin Cuppen

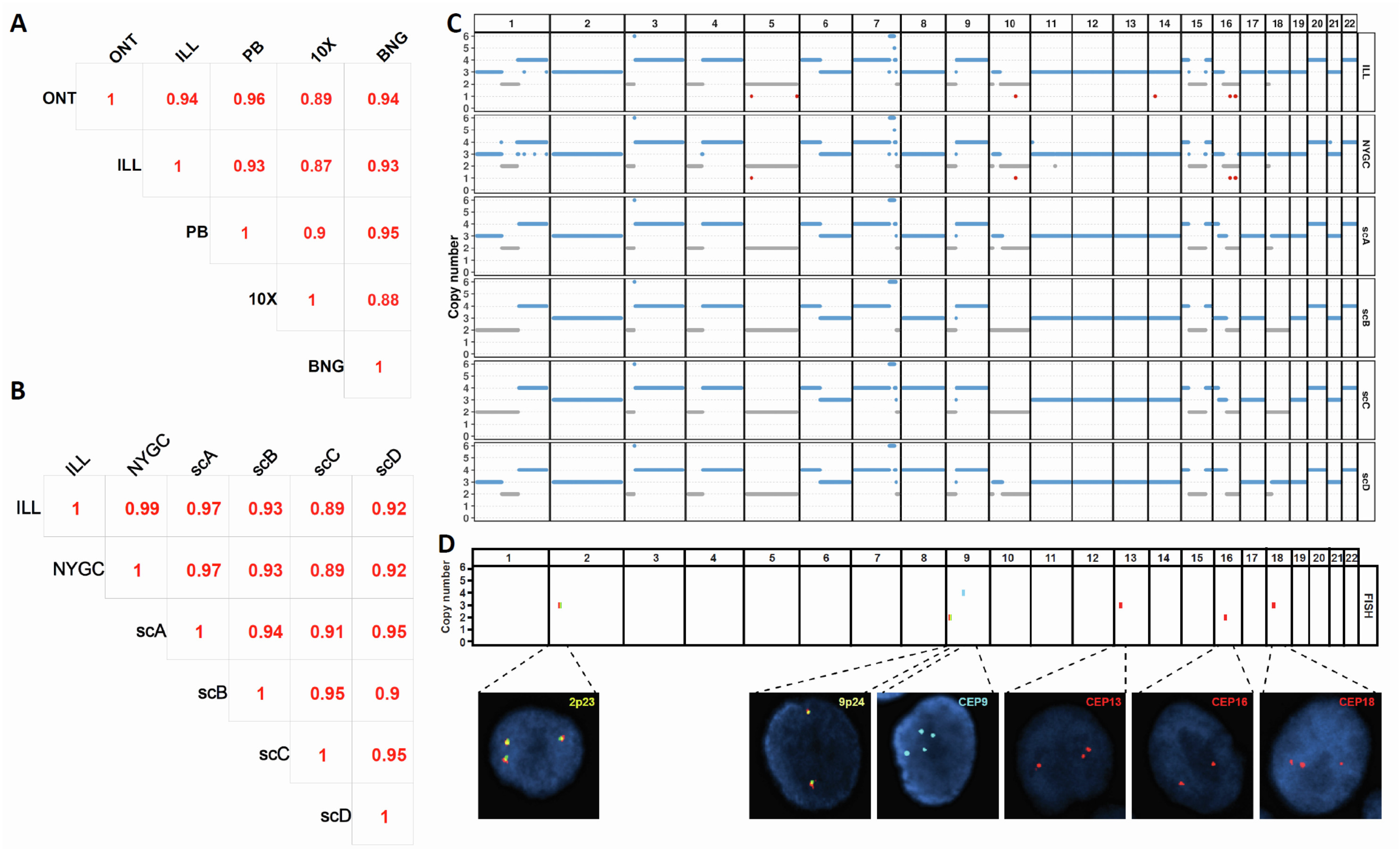

Supplementary Figure 1 - Related to Figure 1: Copy number correlation within our datasets and external datasets.

Correlation index of CNA calls for **(A)** each of the pairwise comparisons of the datasets generated in our study and **(B)** the comparison of our ILL dataset and the external sets from bulk sequencing in NYGC (Arora et al. 2019) and the 4 clusters differentiated by single cell sequencing (scA-D) (Arora et al. 2019; Velazquez-Villarreal et al. 2020). **(C)** Copy number profile of the ILL and the external sets. **(D)** Copy number status of 6 distinct genomic locations as determined by FISH

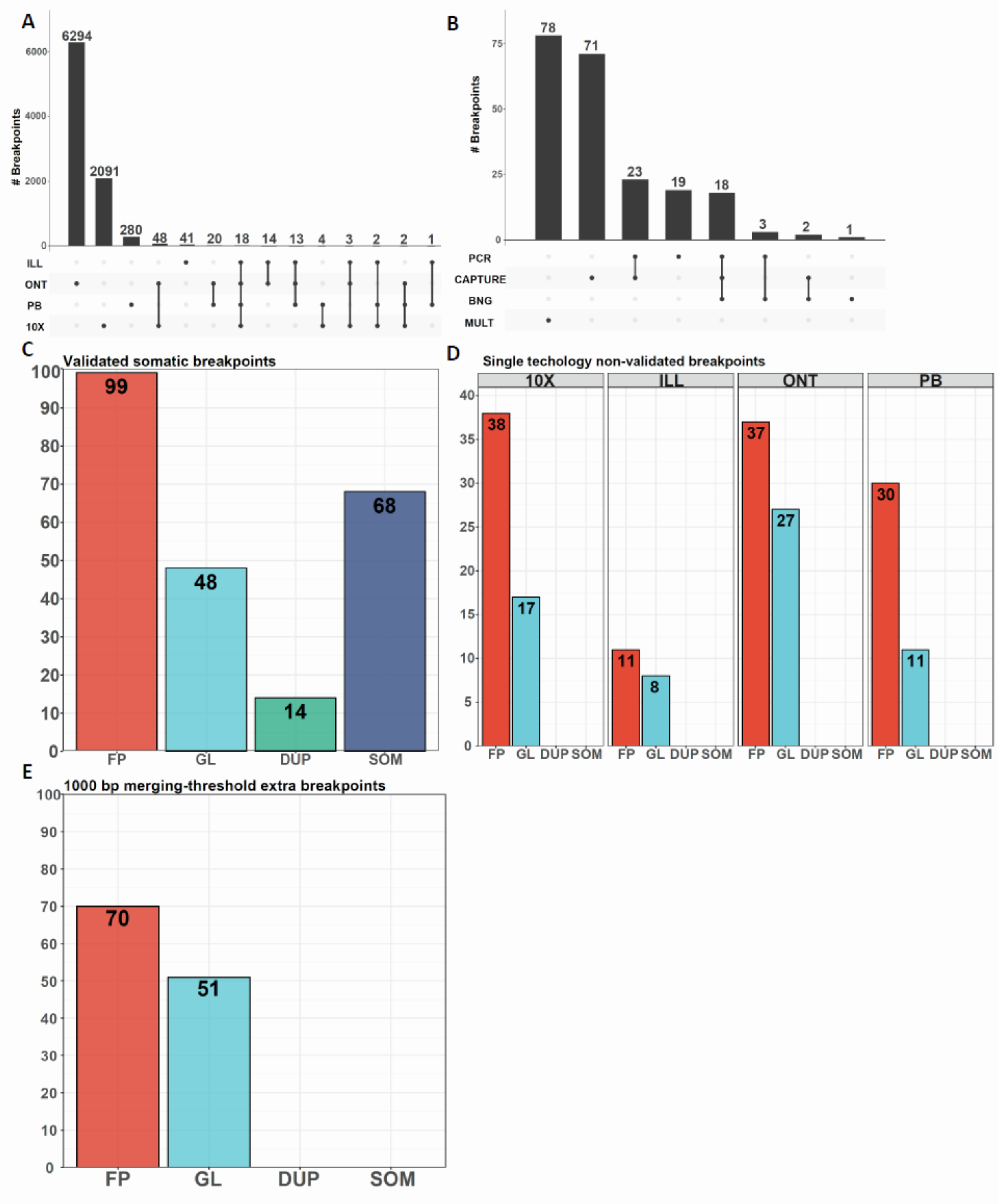

Supplementary Figure 2 - Related to Figure 2: Generation of a somatic SV truth set
(A) Intersection of the total of 8,831 candidate SV calls merged from all platforms used and presence per in the raw call set per technology. **(B)** Number of validated somatic SV calls per validation approach including multi technology support (MULT). Manual curation statistics for **(C)** validated or multi-dataset SV calls, **(D)** non-validated and single-dataset SV calls and (E) additional calls generated using a larger breakpoint-merging threshold. FP = false positive, GL = evidence in germline, DUP = duplication of an already called SV, SOM = real somatic variant.

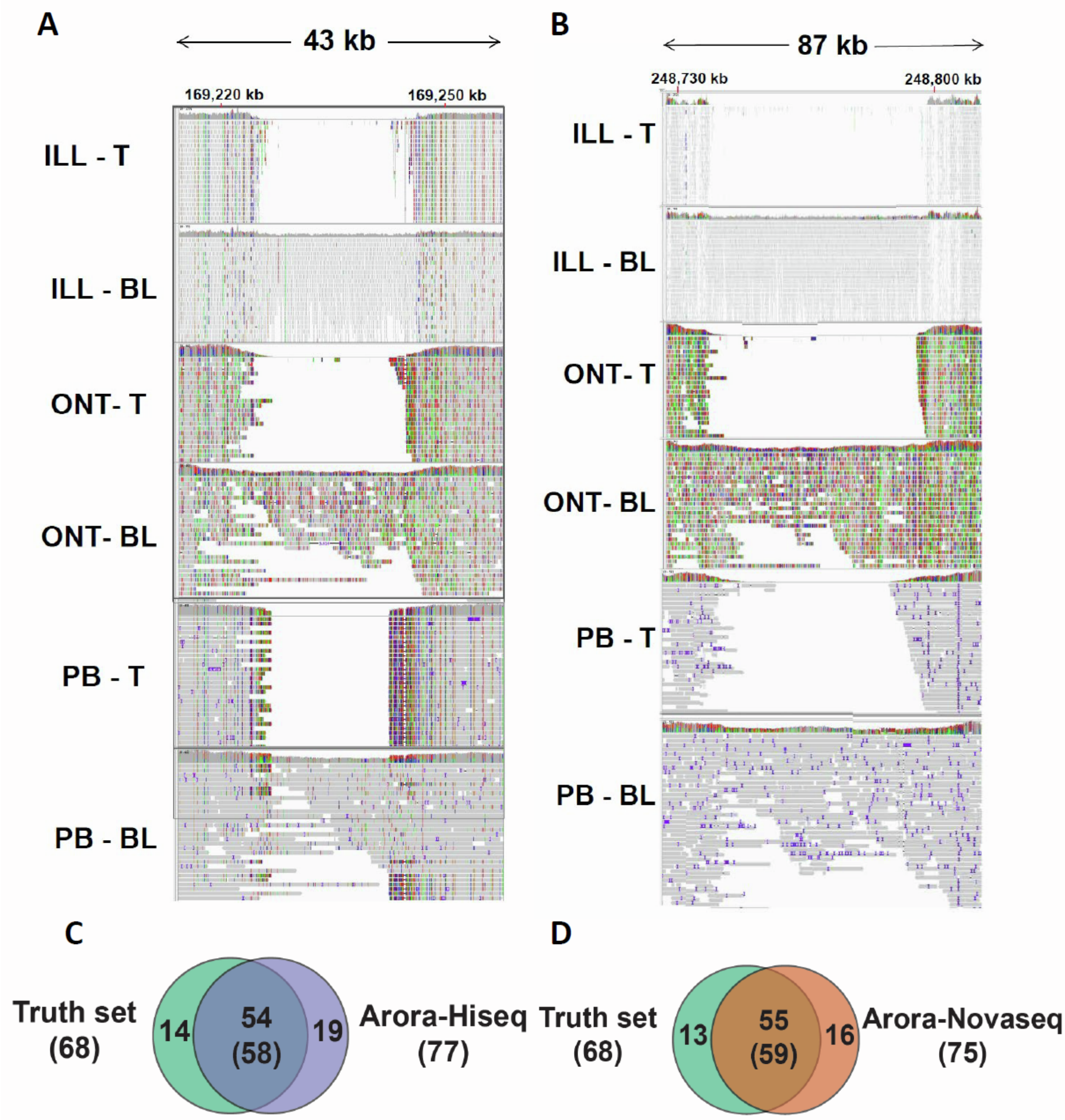

Supplementary Figure 3 - Related to Figure 3: Characterization of the somatic SV truth set
(A, B) IGV screenshots of mapped reads from the ILL, ONT and PB datasets for COLO829 (T) and COLO829BL (BL) of two CNAs on chromosome 1 without associated somatic SVs in the truth set. Overlap of somatic SV calls between our truth set and the two somatic SV sets reported by (Arora et al. 2019)**,** the Hiseq set **(C)** and the Novaseq set **(D)**. One-sided overlaps (i.e. when only one breakpoint of the SV overlaps) are included on the overlap. Numbers in parenthesis indicate the overlap from the Arora set point of view.

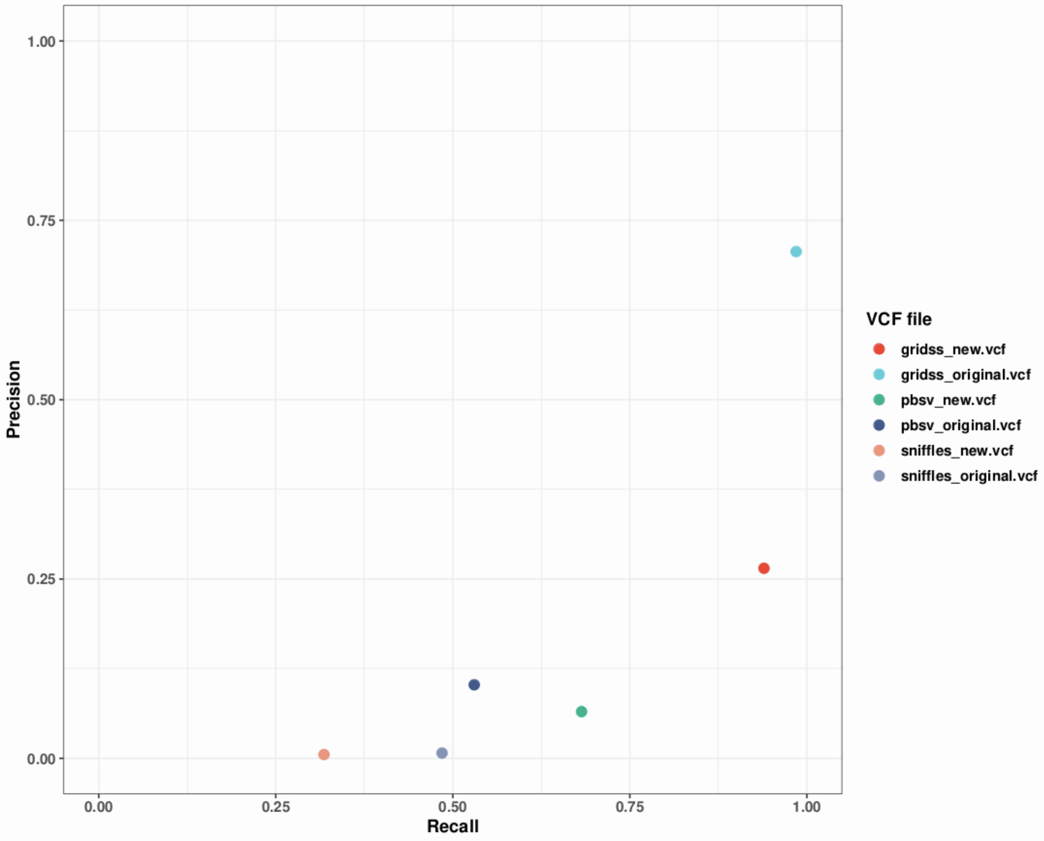

Supplementary Figure 4 – Related to Figure 4: Benchmarking updated SV calling tools using the truth set: Precision vs Recall for the ILL, ONT and PB callsets used in the generation of the truth set and the most recent versions of the same tools at the time of revision.
